# What Patients Value About Reading Visit Notes: A Qualitative Inquiry of Patient Experiences With Their Health Information

**DOI:** 10.2196/jmir.7212

**Published:** 2017-07-14

**Authors:** Macda Gerard, Alan Fossa, Patricia H Folcarelli, Jan Walker, Sigall K Bell

**Affiliations:** ^1^ Department of Medicine Beth Israel Deaconess Medical Center Boston, MA United States; ^2^ Department of Health Care Quality Beth Israel Deaconess Medical Center Boston, MA United States

**Keywords:** patient participation, quality improvement, electronic health records, patient portals

## Abstract

**Background:**

Patients are increasingly asking for their health data. Yet, little is known about what motivates patients to engage with the electronic health record (EHR). Furthermore, quality-focused mechanisms for patients to comment about their records are lacking.

**Objective:**

We aimed to learn more about patient experiences with reading and providing feedback on their visit notes.

**Methods:**

We developed a patient feedback tool linked to OpenNotes as part of a pilot quality improvement initiative focused on patient engagement. Patients who had appointments with members of 2 primary care teams piloting the program between August 2014-2015 were eligible to participate. We asked patients what they liked about reading notes and about using a feedback tool and analyzed all patient reports submitted during the pilot period. Two researchers coded the qualitative responses (κ=.74).

**Results:**

Patients and care partners submitted 260 reports. Among these, 98.5% (256/260) of reports indicated that the reporting tool was valuable, and 68.8% (179/260) highlighted what patients liked about reading notes and the OpenNotes patient reporting tool process. We identified 4 themes describing what patients value about note content: confirm and remember next steps, quicker access and results, positive emotions, and sharing information with care partners; and 4 themes about both patients’ use of notes and the feedback tool: accuracy and correcting mistakes, partnership and engagement, bidirectional communication and enhanced education, and importance of feedback.

**Conclusions:**

Patients and care partners who read notes and submitted feedback reported greater engagement and the desire to help clinicians improve note accuracy. Aspects of what patients like about using both notes as well as a feedback tool highlight personal, relational, and safety benefits. Future efforts to engage patients through the EHR may be guided by what patients value, offering opportunities to strengthen care partnerships between patients and clinicians.

## Introduction

As the trend toward greater transparency accelerates in health care, clinicians with electronic health records (EHRs) and patient portals are inviting patients to view online laboratory results, medication lists, and more recently, visit notes [1-2]. Health care consumers are seeking more data [3], but little is known about their experiences reading and using this information. A better understanding of what motivates patients to interact with their health data may inform efforts that promote patient engagement through patient portals. Thoughtful EHR and patient portal design may be leveraged to strengthen patient and family-centered care and patient-clinician relationships [4-8].

Although clinicians often report negative experiences with the EHR, patient attitudes about the EHR may be more neutral or even positive [9-11]. Greater health information transparency, more rapid communication, patient-friendly educational resources, and easier access to the medical record can send a powerful message of inclusivity to patients and families. What was once the purview of clinicians alone is increasingly shared with patients and families and can lead to better informed shared decision making [12]. Today, over 15 million patients in 40 states have easy access to their visit notes (OpenNotes) through their patient portal [13]. As OpenNotes spreads, sharing health information shows promise not only for patient engagement and adherence [14-16], but also for relational benefits such as enhanced patient trust and satisfaction [17,18].

Even though millions of patients can log on to patient portals to read notes, we understand little about what they value in doing so, perhaps because information sharing has been largely one-way and passive. Opportunities to more effectively connect with various patient populations and family care partners through shared notes are vast, but relatively under-explored [19-22], and patients, families, and communities remain a largely untapped resource as health partners [8]. As patients increasingly gain access to visit notes, they may uncover errors or discrepancies in their records, and they generally lack a systematic way to report this feedback [23]. Tools to guide patients on their health data and systems to efficiently and effectively hear their feedback are needed.

To learn more about the patient experiences with their notes, we piloted an online OpenNotes patient reporting tool as part of a quality improvement initiative [23]. In a 12-month test, we asked patients to report possible inaccuracies in notes. In addition to characterizing patient-identified errors [23], we aimed to understand whether patients thought reading notes and providing feedback was valuable, and if so, why. We envisioned that what patients and care partners value about interacting with their notes could inform organizational patient engagement strategies and further drive patient and family-centered care. This paper focuses on their qualitative responses.

## Methods

### The OpenNotes Patient Reporting Tool

The patient reporting tool was designed together with patients and family members, as well as with Patient Relations and Health Information Management personnel, Patient Safety leadership, clinicians, and other stakeholders. This multidisciplinary team of stakeholders met every other week for nine months to plan the reporting tool and supporting patient education materials, including a patient FAQ specifically designed for the project [23]. These materials underwent several iterations after review by our team, a plain language specialist, and several additional PFAC members who tested the tool and education links. The final patient reporting tool was a 9-item form accessible through a “My Feedback” link located at the end of each visit note. Participants had to first read the note in order to use the reporting tool. Either patients or their care partners (CPs) could complete the form. Questions included whether patients (or CPs) understood the note and care plan, identified possible inaccuracies in the note, had positive feedback for their providers, and found the reporting tool valuable.

Respondents who found the opportunity to read and provide feedback on notes to be “very valuable” or “somewhat valuable” were asked: “What do you like about reading or providing feedback on your note?” We chose this broad exploratory question intentionally because there is little existing data on why patients engage with their health data, how they feel about reading notes, or what benefits they may perceive from a feedback tool linked to their notes. We used this expansive approach because we did not have a preference regarding whether patients responded to their attitudes about reading notes or about using the reporting tool, given that both could inform patient engagement strategies. We anticipated there would be some overlap in responses since patients had to read notes in order to use the tool, but we also hypothesized that some patients may value reading notes alone, and simply use the tool to share this information. Finally, although we considered asking two separate questions, we prioritized streamlining open-ended questions to prevent losing patient interest in completing the form. We anticipated that results from a single exploratory question could then inform more specific future queries as well as targeted interventions to further engage patients and care partners, based on what matters to them the most.

### Participants

All patients with portal access and a visit note by a participating provider during August 2014-August 2015 were invited to participate in the feedback project. Patients received an email notification when a note became available including a link to frequently asked questions (FAQ) [23] and a dedicated email address for any project-related concerns. Patients were told that “The goal (of the project) is to help patients and their providers work together to make sure the information in each patient’s medical record is accurate and care is the best it can be. We also hope to learn what patients like about reading their notes.” Patients were also told that at the end of the QI project, all comments would be de-identified and used to promote organizational learning and quality improvements.

We launched the pilot quality improvement (QI) project with clinicians from 2 of 10 teams in our hospital-based primary care practice. OpenNotes was already implemented at our organization and providers were offered the opportunity to opt-out of participation. As part of the OpenNotes policies at our medical center, clinicians can also “hide” individual notes, such that they do not appear on the portal, although <1% do so (personal communication, Lawrence Markson, MD, Vice President, Clinical Information Systems, BIDMC). All other notes generated by the participating providers included the “My Feedback” link and an invitation for patients to use it.

### Analysis

Two researchers (SKB and MG) independently reviewed and coded a subset of responses to identify common themes. Through discussion, the two researchers merged the themes to develop a codebook, and then coded another subset of responses. Each subset comprised an independent (ie, not previously coded) 10-20% of the data. They repeated this process until no new themes were found. All disagreements were resolved through discussion. Next, the researchers used the codebook to separately code another set of responses and tested reliability between the two researchers (κ=.74). Finally, one researcher (MG) coded the remaining responses using the same codebook.

### Ethics

The proposal for implementation and evaluation of the OpenNotes patient reporting tool was reviewed by our institutional review board and determined to be a quality improvement program. Data collected were integrated into existing QI workflows and used in real time to improve care. Patient participation was voluntary. Patients were told that they, and their provider, might be contacted by Patient Relations personnel if their report pointed to a safety concern. Otherwise, the data populated an aggregate database from which we generated de-identified comments for this analysis. We informed patients that de-identified comments would be used to promote organizational learning and quality improvements. Further details of the methods and patient communications have been published elsewhere [23].

## Results

We analyzed consecutive reports submitted by patients and care partners over the 12 months of the pilot period. In total, 260 reports were submitted; of which, 256 (98.5%) reports indicated that the tool was valuable, and 179 (68.8%) reports included a qualitative response to what patients liked about the OpenNotes reporting tool process. Compared with patients who submitted a report but did not respond to the voluntary qualitative question, patients who provided a response were slightly older; otherwise patient characteristics were not significantly different between the two groups (data not shown). Responses highlighted a total of 8 key themes, presented below. Four themes pertained to what patients value about the content of notes, and the other four described what patients liked about using the reporting tool (for which reading notes was implicit).

### What Patients Value About Note Content

#### Confirm and Remember Next Steps

For many participants, notes served as an extension of the visit. One patient noted:

I sometimes have white coat syndrome where I am a little nervous in the doctor’s office and then cannot remember all that was said. Reading the notes after my visits confirms what I have heard.

By far the most common theme, reading visit notes helped patients to better remember next steps. Many commented on turning to notes as a reminder of tests or other recommended follow-up.

Several participants alluded to the stressful nature of the visit:

I think it is a great way to double check I didn't miss anything if I was not feeling well or was too overwhelmed.

Patients liked reviewing what happened at the visit in the comfort (and pace) of their own homes:

Reading the note takes the burden off of me to remember the details of what we discussed and becomes a useful reference for me.

They also liked the ability to confirm or double-check the doctor’s recommendations independently:

If I forget something, I can go back and read the plan without having to bother the doc[tor].

#### Quicker Access and Results

Patients and CPs valued the opportunity to have access to records and results, stressing the importance of being able to view this information quickly and at any time. Participants found the notes particularly valuable because they provided context. One patient commented:

I like knowing what the results of my tests mean. The records [laboratory results] show the numbers but the notes provide the interpretation in regards to my personal health status.

Participants also liked having longitudinal access to notes, and the benefits of a consolidated reference, “all in one place.” Like an “encyclopedia on a shelf,” OpenNotes provided patients with a cohesive roadmap over the arc of their health journey:

It is now all on record for me to review…and not just after the consult. Allows for history.”

Patients noted a heightened sense of ownership of their records and their health when they could review and interact with their notes collectively and comprehensively over time:

Doctors’ notes are my medical history and until OpenNotes patients had no insight into what is ultimately my medical history.

#### Positive Emotions

Reading notes helped patients gain confidence in their providers, *“confirm[ing] that…care is being handled well.”* It also generated additional positive emotions like hope and encouragement. One patient wrote, *“I like reading my notes because they keep me uplifted.”* Another added, *“I feel less helpless and perhaps more hopeful.”* Participants highlighted the relational benefits of “being heard.” Their comments described a powerful “validation” from reading notes, and feeling listened to and cared for:

We have had a funeral and a hectic week. I felt like someone cared. May seem quite simple but it was a nice human touch. I am a nurse and I am impressed.

#### Sharing Information With Care Partners

The invitation to read notes and provide feedback was particularly appreciated by care partners who support vulnerable patients. In particular, they found notes essential to the coordination of care for their loved ones:

We are grateful to receive “notes” to be able to review the visit and procedures (if any) performed. Especially helpful for older patients who may have hearing and/or some cognitive [or] memory loss.

Patients liked the option to give their note to care partners too: *“I like that I'm able to share how my visit was,”* and *“I can reference info[rmation] to inform my family [and/or] wife [of] what is going on.”* Another patient added, *“I don't have to take tons of notes myself…to make sure I understood.”* OpenNotes connected care partners with information that they may not have otherwise had access to, and provided a way for them to stay updated on medication or treatment plan changes.

### What Patients Value About Their Use of Notes and the Reporting Tool

#### Accuracy and Correcting Mistakes

Patients and care partners commonly noted that what they like about reading notes and providing feedback is the new ability to confirm the accuracy of the note and catch potential errors. As one patient noted, *“I like to see that my medical records as embodied in the notes are consistent with the conversation I have had with my doctor.”* Another noted, *“I appreciate the open exchange and the opportunity to correct any possible misunderstandings.”* Finally, some patients liked testing the accuracy of their own communication, welcoming the opportunity to clarify misunderstandings about their report of symptoms or history through the tool.

[Reading] my notes allows me to see how well I am communicating my issues, which leads to how well my doctors are hearing and documenting my issues. It also allows me to catch errors.

While some clinicians worry that patient-found mistakes may lead to casting blame or trust erosion, several participants explicitly commented on understanding human fallibility and wanting to play a role, alongside their provider, in contributing to note precision: *“It is easy to make a mistake when writing a note. I like that they can be reviewed for accuracy.”* Another added: *“We can work together to make notes accurate, understood, and…a good resource for future medical care.”*

#### Partnership and Engagement

Patients frequently noted that they liked reading notes to *“[Make] sure that we are on the same page,”* and that the feedback tool enhanced a sense of partnership with their clinicians. Participants described notes as a window into how their provider thinks:

I like that level of communication and the ability to see the doctor’s thought process. The more open communication there is, the better care I, as an active participant, have access to.

Patients also saw engagement through OpenNotes and the reporting tool as a two-way street:

Reading the notes can only make me come to my appointments better prepared and help my team understand what issues are important to me and what I need them to hear.

Moving away from the traditional paternalistic view of medicine, the reporting tool encouraged shared agency for health: *“It puts me in an active rather than passive position and cuts out red tape.”* Several responses addressed the value of inviting patients to provide input. One participant noted, *“Health care should be a two-way conversation; this forum provides another opportunity for that.”* Another commented, *“[The note] helps me feel that my [doctor] and I are partners in promoting my health.”*

Finally, several patients and CPs commented on the level of detail, articulation, and precision in the notes. The comprehensive nature of notes helped patients feel that their provider “knows” and cares about them, strengthening a therapeutic alliance through shared values and goals.

#### Bidirectional Communication and Enhanced Education

Patients and CPs often described reading notes as playing a significant role in improving communication between patients and providers, while also increasing learning. As one patient stated, *“It is an opportunity to become more knowledgeable about my condition and how I can manage it better.”* Patients and care partners emphasized the power of print, indicating that some learning styles favor written information, and the importance of an enduring reference: *“I very much appreciate the opportunity to see again in writing what was discussed.”* Patients also reported feeling more informed and gaining a better understanding of their health condition as a result of reading notes, and that the reporting tool extended “teach-back” opportunities from providers to patients, with an opportunity for bidirectional communication:

I like the educational and improvement potential of the process. I learn. My provider learns. All good.

Several reports also emphasized that reading notes and providing feedback affords patients a way to share information without bothering their providers: *“It allows more frequent non-intrusive communication with doctor.”* Patients liked the chance for *“no embarrassing face to face asking of questions if [they] want to understand or know more.”*

#### Importance of Feedback

Patients embraced the opportunity for feedback on many levels: receiving feedback about their health and how they are doing in various aspects of their care, and giving feedback to their providers. Many patients liked the tool because it offered a new way to share positive feedback: *“I appreciate the opportunity to praise my healthcare providers.”* Others saw the tool as a safe haven for feedback:

This is a way to [confidentially] reflect a patient’s reaction to a provider without “causing trouble.” I will use it a lot.

Another noted:

This new project, [OpenNotes] Feedback, is terrific. Finally. Because it is [confidential] I will use it with a mental comfort I have not had till now—over 10 years.

Some patients read notes as a self-feedback mechanism—a way to check how well they were communicating and understood by their providers.

Patients also valued feedback as a way to contribute to the note, for example adding missing information patients found important. Several comments reflected an understanding of quality improvement and a desire to participate in making care better: *“Having the opportunity to provide feedback is important to moving the program forward and helps stimulate innovation.”* Patients appreciated being asked for their input, irrespective of whether they identified a potential safety concern in their note:

I am happy that you asked for feedback—if only so that I can say how helpful it is and how pleased I am to have this site available to me.

As above, patient comments drew a link between the invitation for feedback and the effect of inclusivity on strengthening patient-clinician relationships:

Being able to provide feedback is very important to me as well. I feel it keeps me connected to my health care providers.

## Discussion

### Principal Findings and Implications

With little knowledge on what motivates patients to engage with their health data, we sought to characterize what patients value about reading visit notes as part of a quality improvement initiative. Our findings highlight several insights. Patients and care partners described priorities that can be leveraged to design patient portals that better support patients and families while improving quality of care. For example, participants liked reading notes to remember and confirm next steps. They felt less overwhelmed and more proactive in their care as a result of reading notes. Patients valued the ability to go back to their health information at their own pace and leisure as an enduring, longitudinal resource; open bidirectional dialogue with clinicians and the ability to ask questions with “non-embarrassing” face to face dynamics; and quicker access to notes and results, an established ambulatory care safety priority [20]. Additionally, patients reported developing a greater understanding of their condition from reading notes and liked learning about “the doctor’s thought process.” Taken together, the specific features that patients valued have direct implications for strengthened shared decision making and informed consent [12,24,25].

Participants also particularly valued the ability to check note accuracy and to share notes with family care partners. A feedback mechanism that encourages commentary from patients and care partners, who may catch possible documentation errors or clinically important oversights in the notes, may also improve portals and care. Poor electronic health record interoperability is a recognized problem [26], medication errors are frequent, and missing information poses a safety threat, particularly for vulnerable patients with complex care needs. As supporting family care partners of older or vulnerable patients becomes a health care priority [21], OpenNotes and the reporting tool may empower care partners with health information and provide a space for their feedback. Though some studies question whether patients would be willing to identify errors [27], our findings resonate with recent reports showing that patients and families can recognize quality problems [28,29], and suggest that at least some patients and care partners particularly value working alongside their providers to ensure their records are accurate.

Shifting the nexus of control away from clinicians alone to one that is shared with patients and families and reflects their values has been described in the literature as patient-centered care, person-centered care, and relationship-centered care, among other terms [8,30]. Here, we refer to “patient and family-centered care” although several of the other terms also apply. In our findings, patients suggested that an invitation to read notes and use the reporting tool sends a message of inclusivity and empowerment, validating patients as capable change agents. Such comments resonate with experts’ support for “democratization of health care,” shifting traditional power relationships in medicine, and bringing patient and family voices more consistently to health decisions, system design and patient activation tools so that they can engage in ways that “matter most to them” [8,12]. Inviting patients and families to read notes and give feedback helps to level the playing field, providing more information needed for participation in care. Recognizing that while some patients want to be included in decision-making and treated as experts or safety partners regarding their own experience [31], not all patients desire this degree of engagement [32], and hence the evolution of patient portals should work toward closing the digital divide while respecting individuals’ choices. As information transparency spreads, our findings can help inform patient and family-centered strategies that further engage those patients who seek their health data (Table 1).

Portals and electronic information are never a substitute for meaningful face-to-face time with clinicians. But although doctors worry that computer use during shorter visits can make clinical interactions feel impersonal [33], patients who read notes liked “feeling heard,” describing a deeper sense of caring and respect, and improved patient-clinician relationships. OpenNotes is not a solution for the shortcomings of the EHR, but it may help make the computer feel like less of an obstacle and more of a shared resource, particularly if clinicians turn it toward patients’ view and actively invite them to read notes and even provide feedback after the visit. Although some health care providers worry that doing so may increase liability or erode trust, our findings suggest that this innovation may strengthen partnerships with clinicians, consistent with prior studies and data in other fields suggesting that transparent communication enhances trust [18,34].

The availability of notes may also make face-to-face time more effective. Some patients felt more attentive or present during visits because they didn’t need to take copious notes, knowing they could access the documentation later. Because patients can go back to notes repeatedly and at patients’ own leisure and pace, reading notes may extend the visit, and clinicians may find opportunities to take advantage of this extra “time with patients.” With patients as a consistent audience to notes, clinicians may even begin to adapt note-writing in the future to be more personalized, trust-building, or even therapeutic [35].

**Table 1:** What patients value about OpenNotes: further engaging patients who use the portal.

What patients value			Implications for patient and family-centered quality of care
**Note content**			
	Confirm and remember next steps		Improve adherence and follow up
	**Quicker access and results**		Unburden patients during and after visit, feel less overwhelmed Enable patients to track progress over time; potential for increased “ownership” of healthcare issues (patient accountability) Facilitate patient engagement in diagnostic process
		Easy and long-term access to EHR as a consolidated reference at patient’s own leisure and pace	
	**Positive emotions**		Improve patient experience Foster humanism in patient care
		Encouragement and “whole person” care	
	Sharing information with care partners		Better support care partners with comprehensive clinical information Potentially avert medical errors or preventable readmissions for vulnerable patients due to poor information transfer
**Use of notes and the reporting tool**			
	Accuracy and correcting mistakes		Empower patients to identify and correct documentation errors
	Partnership and engagement		Strengthen patient-clinician relationships including enhanced trust Activate patients in their care Facilitate patient engagement in diagnostic process
	Bidirectional communication and enhanced education		Open transparent dialogue with emphasis on inclusivity Non-intrusive or non-embarrassing way to ask questions Provide enduring resource, and “power of print” for visual learners
	Importance of feedback		Involve patients in QI efforts Create mechanism for positive patient feedback; curb provider burnout

Finally, we were struck by patients’ interest in praising their providers and their description of positive emotions stemming from reading notes. At a time when clinician burnout is in the spotlight [36,37], it is intriguing to consider the potential positive relational effects of OpenNotes on both providers and patients. Creating a space for patients to provide positive feedback for clinicians may bolster morale and even influence positive culture change if amplified across practice settings. Like clinicians, patients and care partners too may be alienated, emotionally distanced and exhausted from interactions with a fragmented and depersonalized health care system [38]. Mechanical, template notes with abundant copy and paste material may exacerbate the problem, and OpenNotes may make this problem more “visible.” On the other hand, restoring some patient narrative to notes may help patients feel heard. Assimilation of multiple visits through integrated note access on a single portal may help unify the patient’s perception of care, particularly if clinicians refer to each other’s notes, as patients learn about how the team works together. Additionally, similar to approaches to decrease burnout for clinicians, enhancing meaningful connections between patients and providers through supportive language in notes and a sense of belonging to the team may be a valuable strategy.

Although these reports reflect the perspectives of patients and care partners who are already engaged by reading notes, organizational exploration of what patients value about note transparency can have a large impact, considering that over 15 million patients have access to their notes across the country today [13]. Building a system in which people want to engage requires knowing what matters most to them. We were struck that half of the themes described by patients reflected what patients valued about reading notes alone, suggesting that simply sharing notes (even without a patient reporting tool) can help patients better remember the care plan, feel less overwhelmed, gain quicker access to results, generate positive emotions, and enable information sharing with care partners. The other themes—ensuring note accuracy, enhanced engagement and partnership, bidirectional communication and education, and the opportunity for feedback and inclusivity—are also valued by patients who read notes, and further strengthened by a patient reporting tool. These can serve as important first steps to inform patient engagement strategies through the patient portal (Table 1). Additional research and health literacy supports are needed to learn what matters most to patients and families who are not yet registered on patient portals and to make that information accessible to them in meaningful ways.

### Limitations

Our findings are limited by the small size of a pilot initiative at a single institution. Respondents likely represent a self-selected population, biased toward activated patients who are registered on the patient portal, use OpenNotes, and are from one geographic area. Patients at our medical center are largely white and more likely to have a 4-year college degree or higher. This quality improvement initiative was designed specifically for one health care organization, limiting generalizability to other patient populations. Although a formal analysis of additional sites is beyond the scope of this report, as the OpenNotes reporting tool has expanded to other clinical settings and organizations, we are seeing similar themes surface, reflecting our findings.

### Conclusion

In summary, as EHR transparency spreads, new ways for patients to engage with their data in ways that matter to them most and to comment on their records are needed. Many aspects of what patients and care partners like about reading notes and providing feedback have important implications for improving patient and family-centered quality of care, safety, and patient-clinician relationships, and can also inform future patient engagement strategies and patient portal design.
